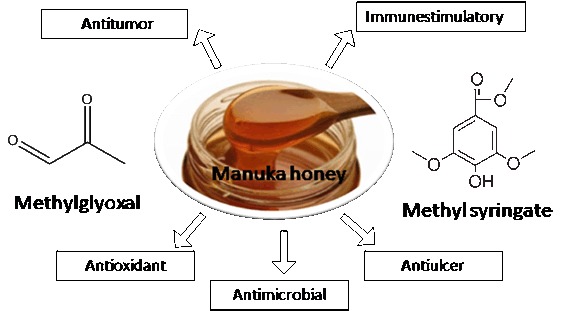# Manuka honey: an emerging natural food with medicinal use

**DOI:** 10.1007/s13659-013-0018-7

**Published:** 2013-07-05

**Authors:** Seema Patel, Simon Cichello

**Affiliations:** 1 San Diego State University, San Diego, CA 92182-7455 USA; 2 School of Life Sciences, La Trobe University, Melbourne, VIC 3086 Australia; 3 Key State Pu-erh Tea Laboratory, Yunnan Agricultural University, Kunming, 650201 China

**Keywords:** manuka honey, methylglyoxal, antimicrobial, wound therapy, anti-ulcer agent

## Abstract

The health value of honey is universally acknowledged from time immemorial. Manuka (*Leptospermum scoparium*) is a tree, indigenous to New Zealand and South East Australia, and from the myrtle family, Myrtaceae. The honey produced from its flowers is a uni-floral honey largely produced in New Zealand. It is becoming increasingly popular as a functional food, seen in the aisles of health stores as its displays superior nutritional and phytochemistry profile over other varieties of honey. Examining existing research databases revealed its biological properties ranging from anti-oxidant, anti-inflammatory, anti-bacterial, anti-viral, anti-biotic and wound healing to immune-stimulatory properties. Methylglyoxal is the unique compound in the honey responsible for some of its potent anti-microbial properties. Further, propolis another component of honey contains chiefly flavonoids (i.e. galangin, pinocembrin), phenolic acids and their esters that may also contribute to its immuno-stimulant properties. Recent findings of the biological roles have been discussed with emphasis on the underlying mechanisms. The hurdles associated in its development as a functional food and also nutraceutical with future scopes have also been mentioned. Relevant data published in MEDLINE, Cochrane library, and EMBASE in the past decade have been gathered to formulate this review.

## Introduction

The health restorative role of honey has been recognized since time immemorial. Even with the current use of synthetic anti-biotic and chemotherapeutic compounds, the use of honey as an anti-microbial and wound management agent has not lost significance. Honey is a supersaturated solution of sugars (i.e. glucose, fructose, sucrose and maltose), which also contains proteins (i.e. pollen), enzymes (i.e. glucose oxidase), amino acids, minerals and vitamins, and is popular as a natural clinical treatment. A broad array of honeys are commercialized (*e.g.* alfalfa, dandelion, clover, apple blossoms, orange blossoms, manuka, heather, gum, neem, acacia and myriad other wildflowers). Manuka honey is distinguished as the most effective medicinal honey, yet remains largely unexplored.

Manuka (*Leptospermum scoparium*) is a shrub belonging to the family Myrtaceae. This plant is a native of New Zealand and South East Australia. The name 'manuka' is derived from the Maori language, the indigenous people of New Zealand. It is an ornamental shrub bearing white, red or pink flowers (Fig. 1). In Southern California, these plants are often found adorning gardens, parks and arboretums. The leaves are often brewed into a tea so it is known as the 'New Zealand tea tree'. Apart from its aesthetic value, this plant has the reputation as a source of healthy mono-floral honey. Bees collect the flower nectar and process it into honey. Most of this honey is produced in New Zealand and then shipped to other countries. The unparalleled health value of this honey is ascribed to it phytochemical content of chiefly methylglyoxal, but also levels of hydrogen peroxide and D-glucono-$\delta$-lactone which are derived from glucose (oxidation) and propolis. Due to its anti-bacterial, wound care, anti-ulcer properties, it is often colloquially known as a 'healing honey'. Manuka honey is renowned for its prolific ameliorative effects on sore throat, burns, infections, gum problem, acne, indigestion; and gastro-oesophageal reflux. This review has been compiled from recent published reports with an aim to disseminate the latest research findings on its importance and to stimulate further research into its use as a functional food and medical applications.

As mentioned, manuka honey displays pathogen inhibition and wound healing actions, evident from the number of research articles comparing it to Portbello honey made in Edinburg (UK), Malaysian tualang honey, Ulmo tree honey from Chile, Greek and Cypriot honeys.[1,2,3,4]

Manuka honey is used both internally and externally. It is marketed as a gel for topical applications (i.e. anti-bacterial, anti-fungal action, wound healing properties), capsules, lozenges, and drinks for oral consumption (presumed immuno-

*To whom correspondence should be addressed. E-mail: seemabiotech83@gmail.com

**Figure 1.** ***Leptospermum scoparium*** **plants abloom in summer, Orange County, California.**

stimulant properties), and often re-blended with other functional compounds like propolis, royal jelly (inherent in the bee hive with raw honey possessing anti-bacterial and immunestimulant properties), olive leaves (*Olea europaea*); presumed hypotensive, and anti-oxidant properties due to iridoid glycosides (i.e. oleuropein), and ginger root (*Zingiber officinale*) extract (presumed to increase in peripheral circulation and anti-inflammatory properties due to pungent principles; i.e. gingerols and shogaols.[5]

It is also used as an ingredient in anti-fungal creams for its clinical effect on ringworm and other fungal pathogens, owing to the organic acid content. It is also added to anti-itch creams mainly for its skin mollifying effect against eczema, rashes, bug bites and poisonous plant contact. A plethora of cosmetic products *e.g.* anti-wrinkle cream, facial cleanser, body lotion, moisturiser, soothing eye gel, lip balm, hand wash and soap contain this honey as a component, presumably for its anti-bacterial and anti-inflammatory properties. Recent developments in clinical use of manuka honey as a topical anti-microbial and wound healing agent, has been discussed to provide information on its clinical scope.

## 1 Bioactive Compounds

Manuka honey has been assessed for its nutritional and phytochemical composition, to predict possible clinical uses. It is rich in glucose oxidase that catalyses glucose to produce hydrogen peroxide ($H_2O_2$) which exerts anti-bacterial properties topically. D-glucono-$\delta$-lactone is also produced which reduces the pH of the honey and in addition to the high sugar osmolarity exerts natural anti-bacterial properties and renders the honey shelf-stable. The low water activity of honey in general (0.6–0.75) also renders it uninhabitable for most micro-organisms.[6] Further, propolis is another nutritionally important component in honey, as it has been reported that *p*-coumaric acid in it up-regulates both detoxification and anti-microbial peptide *in vivo* in bees (*Apis mellifera*)[7]. The level of propolis in the honey varies due to the processing to remove particular matter such as pollen. Propolis also contains galangin, pinocembrin, and phenolic acids which have been shown to display anti-inflammatory effects in asthma, cerebral ischemic protective effects and anti-inflammatory/anti-oxidant properties.[8,9]

Methylglyoxal, the aldehyde form of pyruvic acid is the chief anti-bacterial compound in this honey. Unique manuka factor (UMF) is quality marker to identify and market unadulterated manuka honey. Apart from the methylglyoxal content, methyl syringate, ortho-methoxyacetophenone and 3-phenyllactic acid are other abundant components.[10]

Methyl syringate and its novel glycoside; methyl syringate 4-O-$\beta$-D-gentiobiose (also known as leptosin) are anti-oxidants, commonly found at concentrations between 0.2 to 1.2 $\mu$mol/g honey, and its positively associated with UMF, which is expressed as phenol equivalents of its inherent anti-bacterial activity[11]. High concentrations of 4-hydroxybenzoic acid (4BHA), dehydrovomifoliol and benzoic acid was reported in the honey. 4BHA has been shown to inhibit pancreatic lipases, which are important for the digestion of dietary fats in pigs, whereas benzoic acid and its derivatives display anti-fungal activity to various species, *e.g. Fusarium oxysporum*, which is pathogenic to humans i.e. fungal keratitis.[12,13]

Kojic acid (KA), unedone, 5-methyl-3-furancarboxylic acid, 3-hydroxy-1-(2-methoxyphenyl)penta-1,4-dione, and lumichrome were identified in the honey for the first time. Kojic acid is known to enhance the properties of known anti-fungal agents (i.e. amphotericin B and strobilurin).[15] Dihydroxyacetone is the precursor of methylglyoxal. It was quantified in fresh manuka honey to be between 600 to 2700 mg/kg and 130 to 1600 mg/kg in commercial manuka honey. Methylglyoxal content was measured from 50 to 250 mg/kg in fresh and 70 to 700 mg/kg in commercial manuka honey samples.[16]

## 2 Therapeutic Roles

**2.1 Antioxidant and Anti-inflammatory Activities:** Oxidative stress (*e.g.* cell membrane or DNA damage), is caused by the formation of reactive oxygen species (ROS) such as $O^-$, which are natural by-products of metabolism. The body has a number of defence systems against ROS such as anti-oxidants i.e. endogenous enzymes such as superoxide dismutase, catalase, glutathione peroxidases and exogenous dietary anti-oxidants such as ascorbic acid tocopherols, glutathione. Oxidative stress reduction by manuka honey is well-evidenced. The radical scavenging activity for superoxide ions is attributed to methyl syringate.[17] Further, the flavonoids galangin, pinocembrin, and phenolic acids (i.e. gallic acid) found in the propolis fraction of honey and have known anti-oxidant activities[5]. The anti-inflammation potency of manuka honey was investigated and dose-dependent reduction of *in vitro* human neutrophil superoxide production was reported. Further, in a C57BL/6 J mouse model, the topical application of the honey suppressed ear oedema induced by arachidonic acid.[18] Moreover, internal application possibility of manuka honey against *H. pylori*-induced gastric inflammation was explored. It demonstrated appreciable activity and potentiated the anti-inflammation effect of isothiocyanate-rich broccoli sprouts[19], possibly via reduced cytokine (i.e. macrophage induced IL-8 release) induced ROS formation/ or activity in the stomach mucosal tissue. Furthermore, the anti-inflammatory activity of raw and $\alpha$-cyclodextrin-encapsulated manuka honey was shown to inhibit neutrophil TNF-$\alpha$ secretion, and in-turn reduce inflammation. The anti-inflammation mechanisms require investigation of concerned gene regulation[20]. In a trial comparing the anti-oxidant activity of manuka honey with acacia honey from Germany and wild carrot honey, the former was deemed superior in its phenolic content (899.09 mg gallic acid/kg).[21] It was observed that manuka honey has significantly higher levels of the polyphenols and antioxidant activity than portbello honey (a honey made in Edinburg, UK)[1]

Even though trials have been conducted on total anti-oxidant content of the honey, and respective clinical outcomes, further research needs to be conducted. Determination of the specific therapeutic role of the anti-oxidants and co-factors in manuka honey may reveal synergistic activities. The reduction of inflammation (i.e. cytokine hormones TNF-$\alpha$, IL-6), appears functionally important to support the immune system to defend against microbial infection.[22]

**2.2 Anti-bacterial, Anti-biotic, and Anti-viral Effects:** The broad spectrum anti-bacterial activity of manuka honey has been validated through numerous clinical trials and *in vivo* bacterial challeges ranging from oral infections, dermatitis and skin irritations, intestinal inflammation to nosocomical pathogens.

The capacity of manuka honey with UMF 15 was investigated in reducing dental plaque and clinical levels of gingivitis. Gingivitis is a non-destructive dental disease caused by bacterial plaque and subsequent gum inflammation. The randomized study conducted on 30 subjects for a 21-day trial period showed that there was a significant reduction in the mean plaque scores and the percentage of bleeding sites in the group administered with chewy or liquid manuka honey. This honey may be developed for non-cariogenic confectionery to avert gingivitis and periodontal diseases.[23] In a further study, the effect of manuka honey, chlorhexidine gluconate mouth-wash (chemical anti-septic effective against both Gram positive and negative bacteria) and xylitol chewing gum were compared on dental plaque formation. Sixty healthy subjects received either prophylaxis prior to the study's commencement and then were randomly divided into three groups. After the experimental period of 72 h, the plaque level was measured using the Quigley and Hein plaque index. The statistical tests revealed that the plaque inhibition by manuka honey was similar to that of chlorhexidine mouthwash, which was significantly better than xylitol chewing gum.[24] Moreover, the effect of manuka honey on potentially pathogenic oral bacteria was investigated using the macro dilution broth technique. Manuka showed a total inhibition of multi-species biofilm at the concentration of 200–500 $\mu$g/mL. This honey might be able to reduce oral pathogens within dental plaque,[25] possibly due to $H_2O_2$, as it has been shown previously to display anti-microbial effect against dental plaque at 0.5% concentration with chlorhexidine.[26]

It was reported that dressings impregnated with irradiated manuka honey suppressed staphylococcal infections in hidradenitis suppurativa (a chronic skin disease affecting areas with sebaceous glands), by reducing the time of healing.[27] Moreover, the effect of manuka honey in inhibition of *Staphylococcus aureus* and the mechanism of action were investigated. The bactericidal effects were established by marked structural changes in the honey-treated cells as observed with TEM. The cells were inhibited in completion of the cell cycle (increased number of whole cells with completed septa) as the honey interfered with cell division machinery.[28] Rhinosinusitis is the inflammation of the mucous membrane that lines the paranasal sinuses. *Pseudomonas aeruginosa* and *Staphylococcus aureus* are known to be associated with this pathological condition. The *in vitro* effect of methylglyoxal against both pathogens was determined. The compound proved effective against both planktonic form and biofilm of methicillin-resistant *S. aureus* and *P. aeruginosa.*[29] The effect of manuka honey on *P. aeruginosa* was investigated and an inhibitory efficacy was confirmed. The honey affected the key micro-colony forming genes (i.e. algD and oprF) and causes cell lysis.

The anti-microbial potency (agar incorporation method) and total phenol content (free radical scavenging activity) of several honeys were determined. Manuka honey showed strong efficacy against *S. aureus*. This honey had the highest content of polyphenols with 899 mg gallic acid/kg.[31] The non-peroxide anti-bacterial effects of manuka honey on the proteome of *S. aureus* was determined. The treatment resulted in a significant decrease in the bacterial cell growth rate as well as modulation in gene expression of number of proteins. The honey induced down-regulation of two proteins; dihydrolipoamide dehydrogenase (DLD) and elongation factor Tu (EF-Tu). DLD is a mitochondrial enzyme involved in glycine cleavage system[1] and EF-Tu is part of a mechanism for protein synthesis (i.e. ribosomal protein translation). On the other hand, up-regulation of cold shock protein C (CspC) was observed[32]. CspC is responsible for survival of cells below optimal growth temperatures, a possible signalling pathway for apoptosis in *S. aureus*. Moreover, the *in vitro* effect of manuka honey on *Streptococcus pyogenes* M28 was investigated. Bactericidal effects were found in both planktonic cultures and biofilms, although higher concentrations of honey were needed to inhibit the biofilms. The honey penetrated into a day-old biofilm and interfered with adherence, leading to cell death. The inhibition of binding with the human tissue protein fibronectin was observed and confirmed by a reduction in the expression of genes Sof and SfbI. The promising role of honey in eliminating *S. pyogenes* from wound site and rendering skin graft free from sepsis became evident[33].

The genus *Campylobacter* causes campylobacteriosis, a disease with symptoms including diarrhoea, cramping, abdominal pain, and fever in the infected patients. The anti-microbial effect of manuka honey against a clinical isolate of

*Campylobacter* spp. was reported. The minimum inhibitory concentration (MIC) of the honey against the strains was found to be around 1% (v/v) honey. The low MIC values suggest that honey may still inhibit the growth of the pathogen after dilution by fluid in the gut.[34] In fact, honey requires dilution to raise the pH and thus activate glucose peroxidise, the enzyme that cleaves glucose to yield $H_2O_2$ and D-glucono-$\delta$-lactone.

It was observed that manuka honey is significantly more effective than artificial honey (a mixture of sugars rather than natural honey) against many gut pathogens including *Enterobacter spp*. The antimicrobial action as determined by micro-dilution method established that besides osmolarity, the anti-microbial compounds (i.e. $H_2O_2$, methylglyoxal) are responsible for the observed effect. The honey may be used to inhibit multi-antibiotic resistant microorganisms such as *Salmonella typhimurium* DT104 and extended-spectrum $\beta$-lactamase (ESBL)-producing organisms.[35]

*Stenotrophomonas maltophilia* is often associated with hospital-induced infection. The bacteria colonize breathing tubes and urinary catheters. Manuka honey is effective against this pathogen even at very low MIC. Also, it inhibited *Acinetobacter baumannii*, a serious pathogen with resistance to most antibiotics.[2] Further, manuka honey showed inhibition against several wound bacteria *e.g*. *Bacillus subtilis*, *Escherichia coli*, *Staphylococcus aureus* and *Pseudomonas aeruginosa*. Though not the sole active component, hydrogen peroxide was credited with a partial antibacterial activity.[36,37] For effective wound management, the anti-biofilm effect of manuka honey was investigated against *Proteus mirabilis* and *Enterobacter cloacae* wound isolates. The micro-titer plate assay revealed that the honey at a sub-inhibitory concentration of 10% (w/v) significantly reduced the biofilm development of both isolates. Similarly, at a concentration of 50% (w/v), it caused significant partial detachment of *P. mirabilis* biofilm after 24 h. Treatment of both biofilms with the honey resulted in a significant decrease in colony-forming units per well, with values ranging from 0.35–1.16 and 1.2–7.5 log units, respectively. The anti-biofilm property of the honey was attributed to methylglyoxal.[38]

Currently, anti-biotic use poses a well established impediment, due to the emergence of resistant bacteria. On the other hand, due to multi-phytochemical composition and the array of differing anti-microbial mechanisms, manuka honey seems well presented to inhibit even antibiotic-resistant ‘superbugs’. It was observed that manuka honey and oxacillin interacted synergistically to inhibit methicillin-resistant *Staphylococcus aureus* (MRSA). The honey reversed oxacillin resistance in MRSA and down-regulated mecR1 (a gene that regulates the expression of methicillin resistance).[39] Moreover, the anti-microbial synergistic efficacy of manuka against MRSA and *P. aeruginosa* was evaluated using disc diffusion, broth dilution, E strip, chequerboard titration and growth curves. Five novel anti-biotics (piperacillin/tazobactam, rifampicin, tetracycline, imipenem and mupircoin) and manuka honey combinations were observed to improve anti-bacterial effectiveness *in vitro*.[40] The effect of ‘Medihoney®’ in combination with the widely used anti-biotic rifampicin were investigated. Using checkerboard micro-dilution assays, time-kill curve experiments and agar diffusion assays, a synergism between the two agents was observed against MRSA. The inhibitory principle was suspected to be other than the major component methylglyoxal[41] as catalases protect biofilm against lethal doses of $H_2O_2$[42].

The *in vitro* anti-viral effect of manuka honey on Varicella zoster (a herpes virus that cause chickenpox and the shingles) virus was determined examined. Different concentrations of manuka honey were added to the tissue culture medium of the virus-infected human malignant melanoma cells. This honey showed anti-viral activity against the virus and might be an excellent remedy for zoster rash in developing countries where anti-viral drugs are either expensive or not easily available.[43] The anti-microbial and anti-viral effectiveness of manuka honey has been shown across a range of micro-organisms and viruses mainly due to its broad spectrum of phytochemicals present and their varying degree of mechanisms. This may aid in the anti-inflammatory effects on ulcers and wounds.

**2.3 Anti-ulcer and Wound Healing:** Recurrent wound infections complicate the healing process, and may exacerbate inflammation of the wound site. However, cytokines and other mediators of inflammation may cause ‘out-of-control inflammatory response’ and hamper wound management.[44] Manuka honey has been proven to be an effective agent in healing diabetic wounds, venous ulcers, burns, acne, eczema, psoriasis. The effects of manuka honey dressing on the pH and size on non-healing chronic superficial ulcers were studied. Manuka honey (Apinate® brand name) was applied to wounds for 2 week duration. After the specified time, a significant decrease in wound pH (pH 8 to 7.6) was observed, with every 0.1 pH unit decrease was associated with a 8.1% reduction in wound size. Thus, reduced pH promotes wound healing via increased fibroblast activity, inhibition of protease activity and oxygen release[45]. Moreover, in a randomized control trial, manuka honey improved wound healing in a patient with sloughy venous leg ulcers.[46] The case of a 55-year-old woman with necrotic lesions of the abdominal integuments and the lumbar area following traumatic colonic rupture was reported. When treated with manuka honey wound dressings combined with the GENADYNE A4 negative pressure wound therapy system, it enabled skin auto-grafting on the wound site and facilitate the wound healing.[47] Further, a case study was conducted on a 102-year-old patient with a painful infected venous ulcer complicated by arterial disease. The combination of the superabsorbent KerraMax® (Crawford Healthcare) with the anti-bacterial properties of manuka honey Algivon® (Advancis Medical) created the ideal dressing for the treatment of the mixed aetiology ulcer. It protected the wound site providing an anti-bacterial environment, and inhibiting bacterial growth[48]. In an animal study, the effect of manuka honey gel in ameliorating the wound in the distal limb of a horse was investigated. The treatment using honey and its gel reduced wound retraction and overall healing time compared to untreated control wounds.[49] The case study of leg injury was reported where manuka honey could stimulate production of collagen and thus repaired the connective tissue (i.e. skin). The honey led to complete healing in two weeks. Its efficacy in managing wound healing raises the possibility of its usage in curing Ehlers-Danlos syndrome, a rare inherited disorder characterized by loose joints, fragile skin and blood vessels.[50] Moreover, the efficacy of manuka honey in the treatment of chronic or recurrent pilonidal sinus disease, an abscess near the natal cleft was investigated. Out of the 17 patients who received manuka honey dressing therapy after surgery, 15 achieved complete healing, in a mean time of 65 days.[51] The efficacy of several types of honey, including manuka was investigated in an *in vitro* model of HaCaT (immortal human keratinocyte cell line) re-epithelialization where the epithethial cells re-generate over wound bed. A component of the honey activated cyclin-dependent kinase 2, focal adhesion kinase and rasGAP SH3 binding protein 1, a protein related to apoptosis of damaged and adhesion of new keratinocytes. The wound repair was deduced to be due the activation of keratinocyte re-epithelialization. Manuka honey induced few significant changes compared to other honeys in the expression of epithelial-mesenchymal transition-regulatory genes.[52] This evaluation demonstrated that manuka honey causes a significantly greater reduction of the wound surface area compared to the standard treatment of leg ulcers[53], possibly via methylglyoxal-derived AGE products (i.e. argpyrimidine) which induced NF-κB activation and subsequent apoptosis in lens epithethial cells.[54]

Moreover, the effectiveness of manuka honey-impregnated dressings on the healing of neuropathic diabetic foot ulcers was investigated. A total of 63 patients with type 2 diabetes were randomized to receive either conventional or manuka-soaked dressing. The patients were followed up on a weekly basis for 16 weeks to review the progress of the ulceration. Mean healing time was approximately 31 days for manuka dressing compared to 43 days in conventional dressing. Though the percentage of ulcers healed did not vary significantly between the two groups (97% for manuka dressing and 90% for conventional dressings), the reduction of healing time and rapid disinfection of ulcers by manuka honey treated group were apparent.[55]

The effect of culture media supplemented with a lyophilized preparation rich in growth factors and manuka honey in wound-healing was determined. Human fibroblasts, macrophages, and endothelial cells when subjected to the blend, demonstrated increases in cellular activity in the presence of PRGF, with further increases in activity seen in the presence of PRGF + MH. The fibroblasts proved to be the most positively responsive cells, as they experienced enhanced proliferation, collagen matrix production, and migration into an *in vitro* wound healing model with the supplemented media. Enhancement of dermal regeneration potential was evident.[56] The effect of wound healing may be a culmination of both the anti-inflammatory effect, anti-microbial as well as immuno-stimulatory effect of manuka honey. Immuno-stimulation, in particularly the elevated level of cytokine hormones is merited as one mechanism for wound repair.

**2.4 Immune-stimulatory:** The immuno-stimulatory effect of manuka honey has often been accredited to a number of its phytochemicals; gallic acid, and flavonoids; galangin, pinocembrin. It has been reported that manuka honey increases IL-1$\beta$, IL-6, and TNF-$\alpha$ production from Mono Mac6 cells or human monocytes.[57] On the contrary, cytokine elevation is a by-product of inflammation and cytokines would presumably decrease with consumption of manuka honey as phytochemicals such as gallic acid inhibit inflammation induced NF-κB, interleukin-1$\beta$, and TNF-$\alpha$ expression.[58] Further, both pro-inflammatory and anti-inflammatory effects of manuka honey was observed. The contradictory results of stimulation as well as inhibition of TNF-$\alpha$ was attributed to the formation of AGE products of methylglyoxal which is correlated to systemic TNF-$\alpha$ levels[59,60]. Further, a small molecular weight compound from manuka honey was isolated by filtration followed by reverse-phase solid-phase extraction. The bioactive component stimulated production of TNF-$\alpha$ via TLR4, suggesting it is related to the innate immune system response. Blocking TLR4 significantly inhibited honey-stimulated cytokine production.[61] Also, the ability of manuka honey was assessed in eliciting the release of TNF-$\alpha$ from monocytic cell lines THP-1 and U937 which stimulated TNF-$\alpha$ release from THP-1. Further *in vivo*, rather than *in vitro* cell line research needs to be conducted to confirm the effect of manuka honey on cytokine hormone release from macrophages as a true reflection of immune response.

**2.5 Anti-tumour Potential:** The anti-proliferative activity of manuka honey was investigated on murine melanoma (B16.F1), colorectal carcinoma (CT26) and human breast cancer (MCF-7) cells *in vitro*. The results displayed a time- and dose-dependent effect, with maximal efficacy at 0.6% (w/v). Furthermore, the activation of a caspase 9-dependent apoptotic pathway, led to the induction of caspase 3 which encoded for reduced Bcl-2 expression, DNA fragmentation and final apoptosis.[45] An *in vivo* syngeneic mouse melanoma model was used to assess the potential effect of intravenously-administered manuka honey alone and in combination with paclitaxel. The treatment with honey alone resulted in a 33% inhibition of the tumour growth. However, a better control of tumour growth was observed in animals treated with paclitaxel combined with manuka honey (61% inhibition).[62] Further, gallic acid, an important phenolic acid present in manuka honey has been shown in an alcoholic extract of *Potentilla fulgens* which reduced Ehrlich ascites tumour in mice and MCF-7 cancer cells via induction of apoptosis by the inhibition of the protein survivin and glutathione lowering effect.[63]

It must be stated that cell cultures do not represent a whole living organism, as the effects of digestion, enterocyte and hepatic conjugation of phytochemicals may modify their chemical structure and therefore their effectiveness. Further human trials are needed to confirm the effectiveness of manuka honey phytochemicals both solo and in synergy.

## 3 Mechanisms of Action

The mechanisms of therapeutic actions of manuka honey have been adequately deduced. Many factors have been attributed to its clinical implications, as the honey contains a multitude of phytochemicals. Recalcitrant wounds generally have an alkaline milieu. The bacterial proteases are active in alkaline condition and increase the risk of infection. The acidic pH of manuka honey lowers the alkalinity, thus inactivates the proteases and helps in healing. Further, the honey increases fibroblast activity (stimulated by cytokines) and oxygen release (aerobic condition hampers pathogenic growth), augmenting the remedial process.[45] The acidity of manuka honey may be attributed to the presence of D-glucono-$\delta$-lactone, phenolic and organic acids. D-glucono-$\delta$-lactone is used in food technology as an acidifier with the E number E575, and is partially hydrolyzed in water to its acidic form; gluconic acid. It is suggested that the 'low pH effect' of

| Biological roles | *In vitro* | *In vivo* | Clinical trials | References |
| --- | --- | --- | --- | --- |
| **Antioxidant and anti-inflammatory** | Radical scavenging activity;<br><br>Reduction of human neutrophil superoxide production;<br><br>Enhancement of the antiinflammatory effect of broccoli | | | 17, 18, 19, 20, 21 |
| **Antibacterial and Antibiotic** | Total inhibition of multi-species biofilm;<br><br>Suppression of the staphylococcal infections in hidradenitis suppurativa;<br><br>Interference with cell division machinery of *Staphylococcus aureus*;<br><br>Inhibition of planktonic form and biofilm of methicillin-resistant *Staphylococcus aureus* and *P. aeruginosa*;<br><br>Decrease in the *Staphylococcus aureus* cell growth rate as well as modulation in gene expression;<br><br>Inhibition of *Streptococcus pyogenes*, *Campylobacter* spp24, multi-antibiotic resistant microorganisms such as *Salmonella typhimurium* DT104, *Stenotrophomonas maltophilia and Acinetobacter baumannii*;<br><br>Reduction in the biofilm development of *Proteus mirabilis* and *Enterobacter cloacae*;<br><br>Synergy with antibiotic against MRSA and *P. aeruginosa* | | Reduction in the mean plaque scores and the percentage of bleeding sites | 23, 24, 25, 27, 28, 29, 30, 31, 32, 33, 34, 35, 36, 38, 39, 40, 41 |
| **Antiviral** | Inhibition of varicella zoster virus | | | 43 |
| **Antiulcer and wound-healing** | Wound repair due to the activation of keratinocyte re-epithelialization;<br><br>Enhancement of dermal regeneration with combination to growth factors | Reduction of wound retraction and overall healing time in horse | Decrease in the pH led to reduction in wound size;<br><br>Improvement of wound healing in patients with sloughy venous leg ulcers;<br><br>Enable skin auto-grafting on the wound site;<br><br>Stimulation of production of collagen;<br><br>Helpful in the treatment of chronic or recurrent pilonidal sinus disease;<br><br>Reduction of healing time and rapid disinfection of ulcers neuropathic diabetic foot ulcers | 45, 46, 47, 48, 49, 50, 51, 52, 53 |
| **Immunestimulatory** | Increase in IL-1$\beta$, IL-6, and TNF-$\alpha$ production from Mono Mac6 cells, monocytic cell lines THP-1 and U937 | | | 51, 57, 61 |
| **Antitumor** | Dose-dependent anti-proliferative effect on murine melanoma (B16.F1) and colorectal carcinoma (CT26) as well as human breast cancer (MCF-7) cells i | 33% inhibition of tumor growth in mouse | | 62 |

manuka honey would be due to the gluconic acid released from D-glucono-$\delta$-lactone when it metabolises with aqueous wound exude. When applied topically, honey dries the mucus of skin injury site and renders the wound inhospitable for bacterial proliferation. The viscous honey when slathered on dressing, creates an osmotic pull, that helps the absorption of excess wound exudates, with hydrogen peroxide, methylglyoxal display anti-bacterial effects to inhibit the growth of pathogenic bacteria. Methyglyoxal is toxic towards pathogens even at very low concentrations interrupting cell division, arresting growth, and specifically causing the degradation of bacterial DNA.[64]

## 4 Issues Encountered

Though the benefits of manuka honey have been widely documented, there are instances of adverse effects that warrants thorough safety analysis before mass prescription is undertaken. Methylglyoxal in this honey is responsible for imparting the strong anti-bacterial activity. However, it also serves as precursor of advanced glycation end products (AGEs). Methylglyoxal, along with its derivatives, impair diabetic wound healing by interferring with the target molecules (i.e. forming adducts on proteins and DNAs, the anomalous cross-linking leading to reduced collagen elasticity, enhancing neutrophil activity resulting in inflammation).[65] Also, this glycating agent is associated with increased atherogenicity. It has been observed to modulate low-density lipoprotein into small, dense particles further increasing the cardiovascular risk in diabetics.[66] It binds to proteins that contribute to the morbidity of aging and diabetes mellitus, such as renal diseases.[67] A prospective, controlled study on chinchilla was conducted to determine the ototoxic effects of manuka honey. After 2 weeks of administration into the ears, the chochleae were removed for analysis. At 50% concentration, manuka honey exerted bactericidal properties against biofilms of *Pseudomonas aeruginosa* and *Staphylococcus aureus*. However, this concentration led to facial paralysis, vestibulotoxicity and hearing loss.[68] Often, kanuka honey is traded as manuka honey, which lacks the nutritive quality of the latter.

## 5 Future Directions

Escalated research is required to further analyze the phytochemical spectrum and the pharmaceutical refinement of active constituents in manuka honey. Further verification is required to determine whether geographical region has any impact on the composition of honey. At present, the honey production remains confined to New Zealand though it may be grown in other parts of the world. Currently, manuka plants grow in Southern California and may prove suitable for harvesting quality honey. The possibility of fortifying foods with this honey and therapeutic applications should be explored for possible development of nutraceuticals. For example, the addition of methylgyloxal has been shown in chicken breast patties to reduce the food pathogen *Salmonella typhimurium* and *S. enteritidis*, extending manuka honey's application to food technology and public health.[69] Furthermore, the honey has been suggested to possess anti-fungal property but there is no empirical data to validate the efficacy and thus may be the next crucial step to explore these claims. The presence of kojic, benzoic and gallic acids (GA) in manuka honey suggest that they may be responsible for the anecdotal anti-fungal properties although further research is needed. Investigating its anti-cancer potential with other immuno-modulating and anti-oxidant containing herbal extracts such as Cat's claw (*Uncaria tomentosa*), or Lingzi (*Ganoderma lucidum*) may also be a significance contribution as a combined multi-facet complementary therapy.

## 6 Conclusions

Antibiotic resistance of bacteria is on the rise, thus the discovery of alternative therapeutic agents are justified. The recognition of biological activities of manuka honey is soaring and more research impetus may reveal increased clinical uses. More randomized controlled trials are required to determine the efficacy and safety of manuka honey. Further investigation is warranted to identify the active components and mechanisms responsible for these activities. Given adequate research attention, these phytochemicals can be used more extensively in complementary medicine either as isolated compounds or as whole honey extracts. A range of honey-based products may also be developed to enhance the existing anti-biotic and anti-septic creams, using manuka honey in more readily available forms.

**Open Access** This article is distributed under the terms of the Creative Commons Attribution License which permits any use, distribution, and reproduction in any medium, provided the original author(s) and source are credited.
